# *Bifidobacterium infantis* and *Bifidobacterium breve* Improve Symptomatology and Neuronal Damage in Neurodegenerative Disease: A Systematic Review

**DOI:** 10.3390/nu17030391

**Published:** 2025-01-22

**Authors:** Manuel Reiriz, Ana Isabel Beltrán-Velasco, Víctor Echeverry-Alzate, Esther Martínez-Miguel, Silvia Gómez-Senent, Sara Uceda, Vicente Javier Clemente-Suárez

**Affiliations:** 1 NBC Group, School of Life and Nature Sciences, Nebrija University, 28248 Madrid, Spain; mreiriz@nebrija.es (M.R.); abeltranv@nebrija.es (A.I.B.-V.); vecheverry@nebrija.es (V.E.-A.); emartinezmi@nebrija.es (E.M.-M.); sgomezse@nebrija.es (S.G.-S.); 2 Faculty of Sports Sciences, Universidad Europea de Madrid, Tajo Street, s/n, 28670 Madrid, Spain; 3 Grupo de Investigación en Cultura, Educación y Sociedad, Universidad de la Costa, Barranquilla 080002, Colombia

**Keywords:** neurodegenerative disease, probiotics, Alzheimer’s disease, Parkinson’s disease, neurodegenerative process, *Bifidobacterium infantis*, *Bifidobacterium longum* subsp. infantis, *Bifidobacterium breve*

## Abstract

**Background/Objectives**: This systematic review focused on collecting the most significant findings on the impact of the administration of *Bifidobacterium infantis* (or *Bifidobacterium longum* subps. infantis) and *Bifidobacterium breve*, alone, in conjunction, or in combination with other strains, in the treatment of neurodegenerative diseases including Alzheimer’s disease (AD) and Parkinson’s disease (PD). These diseases are characterized by the progressive degeneration of neurons, resulting in a broad spectrum of clinical manifestations. AD is typified by a progressive decline in cognitive abilities, while PD is marked by motor symptoms associated with the loss of dopamine (DA). **Methods**: Five different databases, ScienceDirect, Scopus, Wiley, PubMed, and Web of Science (WoS), were reviewed and the studies were screened for inclusion by the following criteria: (i) studies that specifically evaluated the use of *Bifidobacterium infantis*, *Bifidobacterium longum* subsp. infantis, or *Bifidobacterium breve* as a therapeutic intervention, either in human or animal models, in the context of neurodegenerative diseases; (ii) the studies were required to address one or more of the pathologies examined in this article, and the pathologies included, but were not limited to, neurodegeneration, Alzheimer’s disease, Parkinson’s disease, and oxidative stress; (iii) the full text was accessible online; and (iv) the article was written in English. **Results**: The data suggest that these probiotics have neuroprotective effects that may delay disease progression. **Conclusions**: This study provides updated insights into the use of these *Bifidobacterium* strains in neurodegenerative diseases like AD and PD, with the main limitation being the limited number of clinical trials available.

## 1. Introduction

Currently, it is known that there are numerous neurodegenerative diseases (more than 100 have been described to date), as well as medical conditions related to neurodegenerative processes [1,2]. These conditions encompass oxidative stress and mild cognitive impairment (MCI), which often co-occur with the aging process. Neurodegenerative diseases also encompass highly prevalent pathologies, including Alzheimer’s disease and Parkinson’s disease [3,4]. The prevalence of Alzheimer’s disease is particularly noteworthy, affecting nearly 47 million people worldwide. Conversely, Parkinson’s disease currently impacts approximately 8.5 million individuals [5].

Oxidative stress, defined as a state of imbalance between the production of reactive oxygen species (ROS) and the body’s ability to neutralize them, is a significant contributor to cognitive impairment by promoting the release of proinflammatory cytokines, such as IL-1β and TNF-α [6,7,8]. This inflammatory and oxidative environment can perpetuate neuronal damage through the alteration of synapses and the accumulation of intracellular aggregates, such as β-amyloid or α-synuclein, which are key characteristics of pathologies such as AD and PD [9,10]. Conversely, MCI is a heterogeneous condition affecting multiple domains of cognitive function, potentially representing an intermediate stage preceding dementia, including the initial symptoms of AD [11,12,13,14].

Alzheimer’s disease is characterized by the buildup of amyloid plaques and neurofibrillary tangles [15,16,17,18,19]. Synapse loss is one of the earliest pathological events in AD, strongly correlating with cognitive decline and impaired memory function [20]. AD’s etiology is characterized by gliosis, which plays a critical role in the disease’s progression along with the activation of astrocytes and microglia that contribute to synaptic dysfunction [21,22]. Current therapeutic interventions for AD prioritize early stage management, encompassing pharmacological strategies to modulate amyloid metabolism, reduce inflammation, and enhance synaptic function, in conjunction with non-pharmacological approaches, such as cognitive training and lifestyle modifications [23,24].

Parkinson’s disease is a progressive neurodegenerative condition characterized by the premature destruction of dopaminergic neurons in the midbrain, impaired communication between the prefrontal cortex and subcortical areas, and damage to the basal ganglia [25,26,27,28]. Pharmacological interventions, including levodopa and dopaminergic agonists, or surgical interventions, such as deep brain stimulation, are employed in PD treatment [29,30].

In recent years, an increasing body of research has identified a link between alterations in the gut microbiota (GM) and the onset and progression of neurodegenerative diseases, including AD and PD [31,32,33]. While GM profiles exhibit significant variability and are influenced by factors such as mode of birth, diet, stress, lifestyle, genetics, and age, it has been observed that certain forms of dysbiosis, or the loss of microbial homeostasis, are associated with these neurodegenerative conditions [34]. This imbalance in the microbial community may influence the disease process through the modulation of endocrine pathways, immune signals, and neurological factors [35,36]. Therapeutic proposals, such as probiotics, seek to intervene in these alterations. However, the efficacy of these interventions varies across individuals due to the heterogeneity of microbiota profiles. The available evidence suggests potential benefits, including the reduction in systemic inflammation, the modulation of gene expression associated with neurodegeneration, and improvements in motor and non-motor symptoms [32,37,38].

Among the most studied probiotics, those composed mainly of *Bifidobacterium* and *Lactobacillaceae* were found. The *Bifidobacteriaceae* genus comprises Gram-positive bacilli, among which the species *Bifidobacterium longum* and *Bifidobacterium bifidum* are notable [39,40], whereas the *Lactobacillaceae* family comprises long, straight, or curved Gram-positive, anaerobic, lactic acid-forming bacilli.

Several studies suggest that the administration of different strains of *Bifidobacterium*, alone or in combination, significantly improve the neuropsychiatric symptoms present in neurodegenerative processes. Thus, studies addressed by Mohammadi et al. [41,42], demonstrated that the probiotic pretreatment composed of a combination of *Lactobacillus helveticus* R0052 and *Bifidobacterium longum* R0175 in a lipopolysaccharide (LPS)-treated rat model resulted in a reduction in hippocampal apoptosis and the attenuation of inflammation and reduced the LPS effects on memory due to the expression of BDNF. In another study, Zhang et al. [43] investigated the capacity of proteins and peptides to function as growth factors for *Bifidobacterium*, emphasizing their capacity to modulate the microbiome. Additionally, Ni et al. [44] demonstrated that treatment with *Bifidobacterium* and *Lactobacillus* improved inflammatory bowel disease in zebrafish by regulating the intestinal barrier and microbiota.

Due to the diversity of comparing the data obtained in studies dealing with the use of *Bifidobacterium infantis*, *Bifidobacterium longum* subsp. infantis, and *Bifidobacterium breve*, it is necessary to review the main research obtained in this area. *Bifidobacterium infantis* has been widely studied for its beneficial effects on gut health, particularly in preterm infants, showing significant promise in reducing gastrointestinal issues [45]. Similarly, *Bifidobacterium longum* subsp. infantis has been noted for its potential in alleviating symptoms of irritable bowel syndrome and other gastrointestinal disorders [46]. In this vein, *Bifidobacterium longum* subsp. infantis has been demonstrated to modulate cytokine synthesis and immune cell function, thereby reducing inflammation and enhancing intestinal functionality. In experimental models of necrotizing enterocolitis, the administration of this probiotic strain has been shown to reduce the incidence and severity of the disease, concurrently lowering inflammatory markers and altering the composition of the GM. Moreover, *Bifidobacterium longum* subsp. infantis has been demonstrated to enhance the intestinal barrier strength, thereby reducing the intestinal permeability and offering protection against bacterial translocation and associated inflammation [47].

The use of metabolomic tools has facilitated the identification of altered metabolic biomarkers, which are pivotal in comprehending the impact of these probiotics. Notably, substantial alterations in amino acids, such as tryptophan, and in specific phospholipids have been observed in the hippocampus of patients with AD. These alterations are directly associated with memory deficits and synaptic dysfunction.

It is important to note that *Bifidobacterium infantis* is also known as *Bifidobacterium longum* subsp. infantis by the scientific community; infantis is often classified as a subspecies of *Bifidobacterium longum* due to the high genetic similarity observed in genome sequencing. Therefore, both terms are used in this study.

This systematic review focuses on analyzing the impact of administering *Bifidobacterium infantis* (or *Bifidobacterium longum* subps. infantis) and *Bifidobacterium breve*, alone, in conjunction, or in combination with other strains, in the treatment of neurodegenerative diseases. The focus will be on metabolic and cognitive modifications in neurodegenerative processes. This review will present the most recent data on the use of these *Bifidobacterium* strains in order to facilitate a more comprehensive understanding of the advancements in this field of research.

## 2. Materials and Methods

### 2.1. Protocol and Registration

The systematic review protocol was registered on 1 June 2024 at Open Science Framework (OSF) prior to the analysis of the extracted data (Identifier: osf.io/8ksuz; Citation: osf.io/fpqgb; Osf_project: https://archive.org/details/osf-registrations-fpqgb-v1).

### 2.2. Literature Search

This systematic review adhered to the PRISMA (Preferred Reporting Items for Systematic Reviews and Meta-Analyses) guidelines [48,49]. We reviewed five different databases (ScienceDirect, Scopus, Wiley, PubMed, and Web of Science (WoS)) to search for articles published between 2000 and 2024. Two different authors of this article (A.I.B.-V. and M.R.) conducted the research in January 2024, reviewed the titles and abstracts, and assessed the retrievable articles for an in-depth analysis. For this systematic review, the search terms used were the following: [*Bifidobacterium infantis* OR *Bifidobacterium longum* subsp. infantis OR *Bifidobacterium breve*] AND [Neurodegenerative disease OR Parkinson Disease OR Alzheimer Disease].

### 2.3. Study Selection

In both types of studies analyzed in this systematic review (human and animal), the studies were screened for inclusion by the following eligibility criteria: (i) studies that specifically evaluated the use of *Bifidobacterium infantis*, *Bifidobacterium longum* subsp. infantis, or *Bifidobacterium breve* as a therapeutic intervention, either in human or animal models, in the context of neurodegenerative diseases, (ii) the studies were required to address one or more of the pathologies examined in this article, and the pathologies included, but were not limited to, neurodegeneration, Alzheimer’s disease, Parkinson’s disease, and oxidative stress, (iii) the full text was accessible online, and (iv) the article was written in English.

The exclusion criteria included the following: (i) studies that primarily centered on the food industry or the utilization of *Bifidobacterium* strains in food products, without an explicit association with neurodegenerative diseases or oxidative stress, (ii) research that did not encompass a direct examination of the therapeutic effects of *Bifidobacterium* strains in neurodegenerative conditions or conditions related to oxidative stress, (iii) articles that were not available in full text or that did not meet the established language requirements, and (iv) studies with an insufficient methodological design to evaluate probiotic interventions in the selected pathologies.

**Figure 1:**
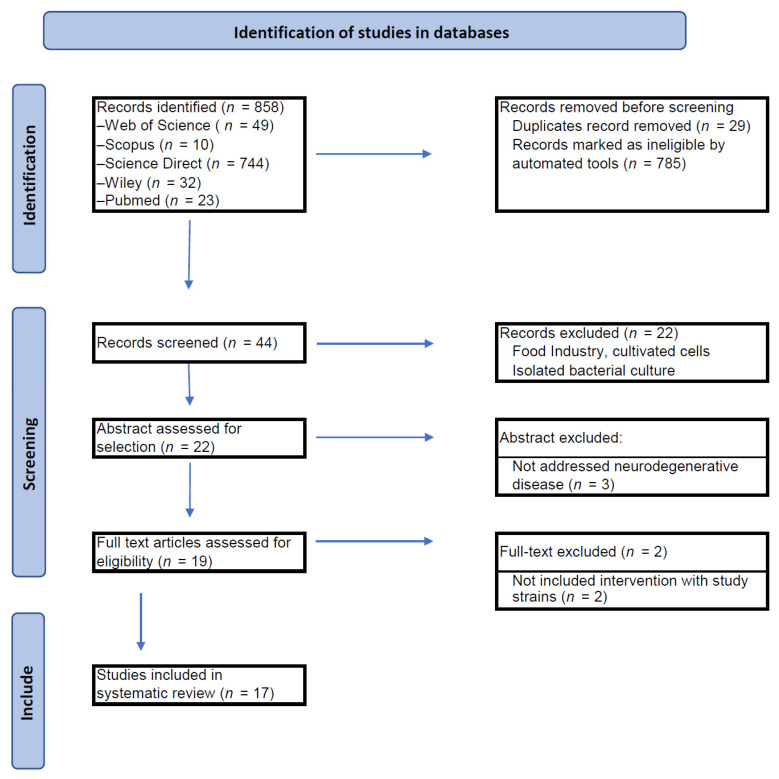
Flow diagram of the search and selection process. A total of 17 studies met the eligibility criteria and investigated the effects of *Bifidobacterium infantis* or *Bifidobacterium longum* subsp. infantis or *Bifidobacterium breve* alone or in combination on neurodegenerative disease (5 studies in human and 12 studies in animal model).

A total of 858 articles were obtained (Figure 1), although only 19 articles were recognized as fulfilling the eligibility criteria. After the duplicates were eliminated, the articles (titles and abstracts) were studied for eligibility. Those that did not include intervention with study strains of *Bifidobacterium* (*n* = 2) were excluded. After the screening phase, 17 studies were selected for comprehensive review in accordance with the predetermined inclusion criteria. Following this process, all the full texts of the selected studies were retrieved in accordance with the established inclusion criteria.

### 2.4. Data Extraction

After the article selection, the studies included were analyzed to extract the data in order to perform a narrative analysis, including a description of the risk of bias. The data extraction was conducted following the next steps (Table 1):

- Study type (differentiating between an animal or a human model).
- Set of strains used in different probiotics (*Bifidobacterium infantis* or *Bifidobacterium longum* subsp. infantis or *Bifidobacterium breve*, alone, in conjunction, or in combination).
- Pathologies associated with neurodegenerative processes, which were included in the study.
- Description of the population.
- Type of methodology that the article employed.
- Intervention parameters (dosage and time of administration).
- Possible results registered after the use of different probiotics (*Bifidobacterium infantis* or *Bifidobacterium longum* subsp. infantis or *Bifidobacterium breve* alone, in conjunction, or in combination).

After the data extraction, a narrative analysis with a description of the risk of bias was conducted.

**Table 1:** Effects of *Bifidobacterium infantis*, *Bifidobacterium longum* subsp. Infantis, and *Bifidobacterium breve*, in isolation or in conjunction with specific strains of these or other species, on neurodegenerative disease symptomatology (in animal and human studies).

	Probiotics	Population	Methodology	Intervention	Results	References	
	Alzheimer’s Disease						
HUMAN STUDIES	Mixture probiotic: *Bifidobacterium longum* subsp. infantis BLI-02, *Bifidobacterium breve* Bv-889, and *Bifidobacterium animalis* subsp. lactis CP-9	40 patients	Probiotic vs. Sham	Probiotic (1 × 10^10^ CFU/capsule for 12 weeks)	In the treatment group, BDNF levels rise significantly from a baseline measurement of 7115.1 ± 4461.9 pg/mL to 9678.5 ± 6652.9 pg/mL at the study’s end (*p* = 0.005). Furthermore, the fold change in cortisol reduction was significantly greater in the treatment group compared to the active control group (119.4% vs. 94.3%; *p* = 0.039).	[50]	https://doi.org/10.3390/nu16010016
	MCI (Mild Cognitive Impairment)						
	*Bifidobacterium breve* A1	117 patients	Probiotic vs. Sham	*Bifidobacterium breve* A1 (2 capsules daily > 1 × 10^10^ CFU) for 12 weeks	A notable distinction was observed between the *B. breve* A1 and placebo group in the subscale of immediate memory on the RBANS and MMSE total score. This was evidenced by a statistically significant difference at the *p* < 0.05 level between the treatment and placebo group at the baseline examination and at the *p* < 0.05 level between the treatment and placebo group at the 12-week examination. However, no significant intergroup difference was observed in terms of changes in scores from the baseline scores.	[51]	https://doi.org/10.3920/BM2018.0170
	*Bifidobacterium breve* MCC1274	115 patients	Probiotic vs. Sham	*Bifidobacterium breve* MCC1274 (A1) (2 × 10^10^ CFU) daily for 24 weeks	A significant intergroup difference was observed in the changes from the baseline of GM (gray matter atrophy in the whole brain) extent score (*p* = 0.013).	[52]	https://doi.org/10.3233/JAD-220148
	*Lactobacillus plantarum* BioF-228, *Lactococcus lactis* BioF-224, *Bifidobacterium lactis* CP-9, *Lactobacillus rhamnosus* Bv-77, *Lactobacillus johnsonii* MH-68, *Lactobacillus paracasei* MP137, *Lactobacillus salivarius* AP-32, *Lactobacillus acidophilus* TYCA06, *Lactococcus lactis* LY-66, *Bifidobacterium lactis* HNO19, *Lactobacillus rhamnosus* HNO01, *Lactobacillus paracasei* GL-156, *Bifidobacterium animalis* BB-115, *Lactobacillus casei* CS-773, *Lactobacillus reuteri* TSR332, *Lactobacillus fermentum* TSF331, *Bifidobacterium infantis* BLI-02, and *Lactobacillus plantarum* CN2018	42 patients	Probiotic vs. Sham	Mixture probiotics (>2 × 10^10^ CFU/g) daily for 12 weeks	After the treatment, an improvement in sleep quality and cognitive function was observed. MMSE (24.75 ± 2.47); Scale-MoCA (22.05 ± 2.14 vs. 20.10 ± 1.45); and Index-PSQI (5.35 ± 2.78 vs. 8.40 ± 1.76; *p* < 0.001).	[53]	https://doi.org/10.1016/j.gerinurse.2023.03.006
	Oxidative Stress						
	VSL#3^®^—*Bifidobacterium infantis* DSM 24737, *Bifidobacterium longum* DSM 24736, *Bifidobacterium breve* DSM 24732, *Lactobacillus acidophilus* DSM 24735, *Lactobacillus delbrückii* ssp. bulgaricus DSM 24734, *Lactobacillus paracasei* DSM 24733, *Lactobacillus plantarum* DSM 24730, and *Streptococcus thermophilus* DSM 24731	62 patients	Probiotic vs. Sham	VSL#3^®^ supplementation (2 capsules daily) for 56 days	Cholesterol or glucose was not affected by Arm B. However, Arm B did lead to a reduction in ESR (*p* = 0.05) and an increase in serum folate (*p* = 0.007) and serum B12 (*p* = 0.001), accompanied by a decrease in the level of homocysteine in plasma (*p* < 0.001). Additionally, the administration of a Sham intervention and a diet supplemented with VSL#3^®^ were associated with an increase in glutathione-S-transferase activity.	[54]	https://10.1016/j.clnu.2014.09.023
	Alzheimer’s Disease						
ANIMAL STUDIES	*Bifidobacterium breve* A1	Male 10-week-old ddY mice	Probiotic vs. Sham	*Bifidobacterium breve* A1 (1 × 10^9^ CFU in 0.2 mL, starting 2 days before Aβ injection) daily for 10 days	*B. breve* A1 has been demonstrated to prevent Aβ-induced cognitive dysfunction, to suppress Aβ-induced changes in gene expression in the hippocampus, and to enhance behavioral impairments when used in conjunction with acetate (*p* < 0.05, *p* < 0.01 vs. control (sham). *p* < 0.05, *p* < 0.01 vs. Aβ (+)).	[55]	https://doi.org/10.1038/s41598-017-13368-2
	Mixture probiotic: *Lactobacillus reuteri*, *Lactobacillus rhamnosus*, and *Bifidobacterium infantis*	50 male Wistar rats	Probiotic vs. Sham	Mixture probiotics 2 g (10^10^ CFU) daily for 10 weeks	The Aβ-treated group exhibited a longer latency period than the control and Sham groups during the MWM training phase (*p* < 0.001). The administration of probiotics was observed to enhance spatial memory and learning abilities, as compared to the Aβ-treated group (*p* < 0.01). Furthermore, the administration of a probiotics mixture was found to result in a notable reduction in MDA levels, as compared to the Aβ-treated group (*p* < 0.001).	[56]	https://doi.org/10.29252/ibj.24.4.220
	*Bifidobacterium breve*—five strains (*B. breve* NMG, *B. breve* MY, *B. breve* CCFM1025, *B. breve* XY, and *B. breve* WX)	63 male C57BL/6J mice (8 weeks old)	5 individual strains of *Bifidobacterium breve*	*Bifidobacterium breve*-5 strains (*B. breve* NMG, *B. breve* MY, *B. breve* CCFM1025, *B. breve* XY, and *B. breve* WX) (1 × 10^9^ CFU/mL) daily for 6 weeks	Administration of *Bifidobacterium breve* NMG and CCFM1025 has shown a significant improvement in behavior impairment and an increase in arm entries, while the other strains did not enhance working memory. CCFM1025, XY, and WX to Aβ_1–42_-treated mice have shown a reduction in the accumulation of the Aβ_1–42_ in the hippocampus (control vs. model: *p* < 0.05 by unpaired Student’s *t*-test; *p* < 0.05 and *p* < 0.01 by one-way ANOVA for all groups).	[57]	https://doi.org/10.3390/nu13051602
	*Bifidobacterium breve* MCC1274	40 C57BL/6J mice (2 months old)	Probiotic vs. Sham	*Bifidobacterium breve* MCC1274 (1 × 10^9^ CFU/mL) five times/week for four months	It is shown that the comparison between Sham and probiotic treatment has revealed a significative reduction in the Aβ_1–42_ concentration in the experimental group. Moreover, the levels in the hippocampus of p-Akt and p-GSK-3β were increased in the treat group vs. the Sham group (*p* < 0.05). Finally, the probiotic used showed an increase in syntaxin level along with an increase in SYT and PSD-95 levels in the hippocampal extracts (*p* < 0.01).	[58]	https://doi.org/10.3390/nu14122543
	*Bifidobacterium breve* A1	52 App knock-in (KI) mice (AppNL-G-F) (3 months old)	Probiotic vs. Sham	*Bifidobacterium breve* A1 (1 × 10^9^ CFU in 0.2 mL, starting 2 days before Aβ injection) daily for 10 days	The probiotic group exhibited a significantly increased exploration time for the novel object compared with the familiar object. Additionally, the discrimination index (DI) was higher in the probiotics group than in the Sham group. *B. breve* MCC1274 supplementation has been demonstrated to suppress Aβ fibril formation. Furthermore, significantly upregulated ADAM10 and PS1 in the hippocampus was observed, whereas AβPP and BACE1 levels did not change (*p* < 0.05).	[59]	https://doi.org/10.3233/JAD-215025
	Vitalon Probiotics (VP) powder: *Bacillus natto*, *Bacillus coagulans*, *Lactobacillus casei*, *Lactobacillus acidophilus*, *Bifidobacterium longum*, and *Bifidobacterium breve*	9–15 mice/group- APP transgenic mouse line J20 & Wild-type (WT) littermate mice (control)	Prebiotic vs. Synbiotic	3.6 g/kg/day prebiotics (WT/P and APP/P)-2.5% inulin; or 4.1 g/kg/day synbiotics (WT/S and APP/S)-Vitalon, intragastrically for 2 months	The probiotic used showed a significant reduction in the level of Aβ_1–4_ (APP/S vs. APP/C); the treatment with the symbiotic leads to a TNF-α level reduction (*p* < 0.05; *p* < 0.001; and *p* < 0.0001).	[60]	https://doi.org/10.1002/iub.2589
	*Bifidobacterium breve* CCFM1025	40 male adult C57BL/6J mice (8 weeks old)	Probiotic + EE vs. Probiotic	*Bifidobacterium breve* CCFM1025 (1 × 10^9^ CFU/mL) + daily for 6 weeks vs. *Bifidobacterium breve* CCFM1025 (1 × 10^9^ CFU/mL) daily for 6 weeks	The use of *Bifidobacterium Breve* with an enrichment environment resulted in an improvement in the working memory and a decrease in the Aβ_1–42_ accumulation (control vs. model: *p* < 0.05, *p* < 0.01, *p* < 0.001, and *p* < 0.0001).	[61]	https://doi.org/10.3389/fimmu.2022.1013664
	*Bifidobacterium breve* CCFM1025	24 male adult C57BL/6J mice (8 weeks old)	Probiotic vs. Sham	*Bifidobacterium breve* CCFM1025 (5 × 10^9^ CFU/mL) vs. Veh-sterile 10% skimmed milk	The treatment with *Bifidobacterium breve* CCFM1025 restored the tryptophan and L-tyrosine levels (*p* < 0.05); several metabolites were modified in the hippocampal tissue in the mice model after the probiotic use (*p* < 0.05); and this probiotic has restored the L-glutamine and serum metabolite phenylalanine hippocampal levels (*p* < 0.05; *p* < 0.01).	[62]	https://doi.org/10.3390/nu14040735
	*Bifidobacterium breve* HNXY26M4	Male mice (16 weeks old)	Probiotic vs. Sham	*Bifidobacterium breve* HNXY26M4 (1 × 10^9^ CFU/mL) daily for 12 weeks	*Bifidobacterium breve* HNXY26M4 treatment has shown a decrease in Aβ_1−42_ levels and reduced the neuroinflammation, synaptic impairment, and oxidative damage in the mice brain. Moreover, the acetate and butyrate levels were increased when the mice were treated with *Bifidobacterium breve* HNXY26M4. (*p* < 0.05, *p* < 0.01, and *p* < 0.001).	[63]	https://doi.org/10.1021/acs.jafc.3c00652
	*Bifidobacterium breve* HNXY26M4	40 male adult C57BL/6J mice (8 weeks old)	Probiotic + EE vs. Probiotic	*Bifidobacterium breve* HNXY26M4 (1 × 10^9^ CFU/mL) daily + EE vs. *Bifidobacterium breve* HNXY26M4 (1 × 10^9^ CFU/mL) daily for 6 weeks	Mice that received only EE (AD^EE^) or EE combined with *B. breve* treatment (AD+BB^EE^) had significantly lower concentrations of Aβ_1–42_ in the hippocampus than AD^SE^ mice (*p* < 0.05, *p* < 0.01, and *p* < 0.001).	[64]	https://doi.org/10.26599/FSHW.2022.9250084
	Parkinson’s Disease						
	*Bifidobacterium breve* A1-MCC1274	156 male C57BL/6 mice (7–8 weeks old)	Probiotic vs. Sham	*Bifidobacterium breve* MCC1274 (A1) (1 × 10^9^ CFU/mL) daily for 4 days	The use of *Bifidobacterium breve* A1 produced a decrease in the spine density reduction that was observed in the PD group, maintaining a density similar to the control group (*p* < 0.01 vs. Control + Saline and *p* < 0.05: between MPTP + Saline and MPTP + *B. breve* A1). However, this probiotic has not shown any beneficial effect in the levels of cAMP in the hippocampus.	[65]	https://doi.org/10.3390/biomedicines9020167
	*Bifidobacterium breve* CCFM1067	40 male C57BL/6 mice (6 weeks old)	Probiotic vs. Sham	*Bifidobacterium breve* CCFM1067 (10^9^ CFU/200 μL saline) daily on days 8–41	The use of *Bifidobacterium breve* CCFM1067 improved the motor deficits produced by MPTP as occurred with the L-DOPA treatment (F (2,21) = 56.94, *p* < 0.0001), NBT (F (2,21) = 33.72, *p* < 0.0001), and RTR (F (2,18) = 21.99, *p* < 0.0001); moreover, this probiotic prevented the increase in TNF- α levels in the striatum (F (2,9) = 91.49, *p* < 0.0001), IL-1β (F (2,9) = 29.53, *p* < 0.0001), and IL-6 (F (2,9) = 24.61, *p* = 0.0002).	[66]	https://doi.org/10.3390/nu14214678

## 3. Results

### 3.1. Quality of Included Studies

**Figure 2:**
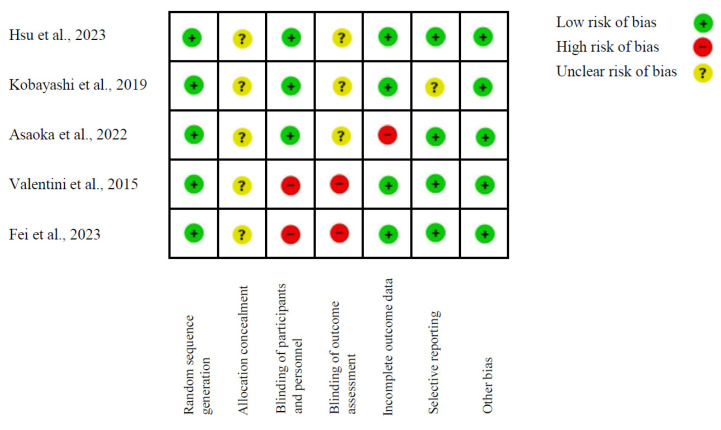
Cochrane Collaboration’s tool for assessing risk of bias (adapted from Higgins and Altman [67]) [50,51,52,53,54].

In the analysis of the risk of bias in human studies, the Cochrane Collaboration tool was utilized (adapted from Higgins and Altman) [67], which provides a systematic approach to assessing the risk of bias in randomized controlled trials, addressing several key domains (Figure 2). Generally, the majority of the included studies exhibited a low risk of bias in several of these domains, thereby enhancing the reliability of the results obtained. However, certain studies, such as Valentini et al. (2015) [54], exhibit some risk of bias in pivotal domains, including allocation concealment, blinding of participants and staff, and selective outcome assessment. These factors have the potential to compromise the study’s internal validity, thereby casting doubt on the objectivity of the conclusions drawn.

Conversely, the studies by Kobayashi et al. (2019) [51] and Asaoka et al. (2022) [52] exhibit unclear risk of bias in several domains, particularly in allocation concealment and blinding of both participants and staff. While these studies provide valuable results, the absence of clarity in these methodological aspects suggests the potential introduction of biases that could affect the interpretation of the observed effects, particularly in terms of clinical and cognitive outcomes. These high or unclear risks underscore the need for greater transparency and methodological rigor in future research, especially in allocation procedures and in the implementation of strategies to minimize observational bias.

**Figure 3:**
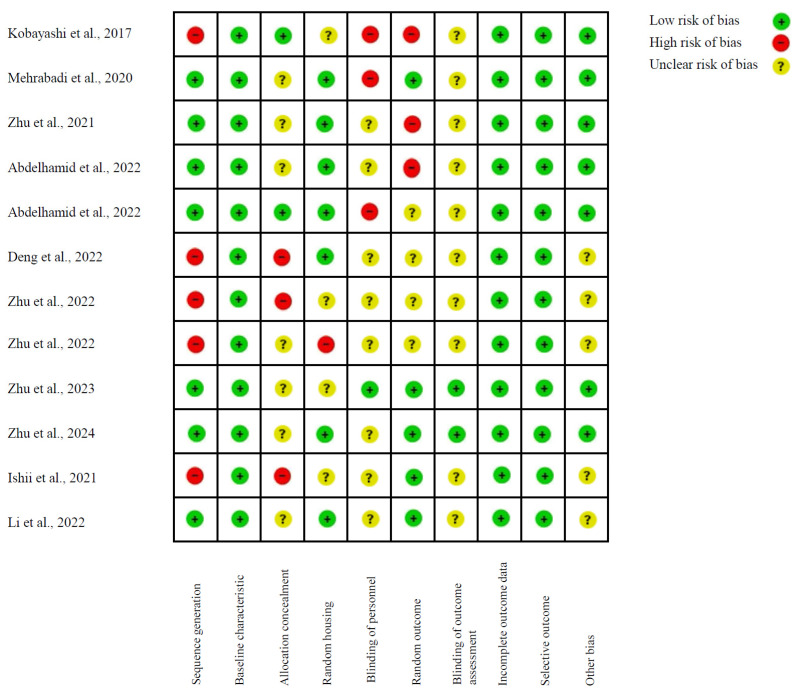
SYRCLE’s tool for assessing risk of bias [55,56,57,58,59,60,61,62,63,64,65,66,68].

For studies employing animal models, the SYRCLE (Systematic Review Centre for Laboratory Animal Experimentation) tool was utilized (Figure 3), having been developed for the purpose of evaluating the risk of bias in animal research. This tool incorporates elements of the Cochrane Collaboration’s risk of bias assessment, with aspects relevant and specific to preclinical studies being integrated. The analysis performed yielded a diverse risk of bias profile among the studies evaluated. While a considerable proportion of studies exhibited a low risk of bias across multiple domains, notable variations were observed in the implementation of critical aspects, such as allocation concealment, randomized animal housing, and the blinding of personnel and outcome assessment. It is particularly concerning that a number of studies demonstrated a high or unclear risk of bias in these areas, which has the potential to compromise the internal validity of the results. These concerns underscore the pressing need to enhance methodological rigor, particularly concerning randomization and blinding techniques, to ensure the reliability of findings in future preclinical research endeavors. Enhanced transparency in the methodology and effective control of these biases could contribute substantially to the enhancement of the quality of evidence generated in animal model studies.

### 3.2. Human Studies

Five studies were conducted in humans. One addressed Alzheimer’s disease and the impact of the use of *Bifidobacterium infantis* or *Bifidobacterium longum* subsp. infantis or *Bifidobacterium breve* in AD patients [53] (*n* = 1); four of them analyzed MCI or oxidative stress in neurodegenerative processes [51,52,54,68] (*n* = 4).

#### 3.2.1. Alzheimer’s Disease

Hsu et al. conducted recent research in 2023 in which they addressed the role of a probiotic composed of *Bifidobacterium longum* subsp. infantis BLI-02, *Bifidobacterium breve* Bv-889, Bifidobacterium animalis subsp. lactis CP-9, *Bifidobacterium bifidum* VDD088, and *Lactobacillus plantarum* PL-02 in AD treatment (1 × 10^10^ CFU/capsule). A total of 40 patients participated in this study, and after 12 weeks of intervention with this probiotic mixture, an increase of 36% in the BDNF (* *p* = 0.005) and in the SOD (antioxidant superoxide dismutase) (* *p* = 0.012) levels was observed in the experimental group vs. the control group. Furthermore, a significant correlation was identified between the increase in BDNF and the improvement in the Mini-Mental State Examination (MMSE) and Montreal Cognitive Assessment (MoCA) evaluations, suggesting a synergistic effect between neurotrophic modulation and antioxidant capacity. Conversely, a decrease in IL-1β (* *p* = 0.041) with a significant reduction in cortisol levels (119.4% vs. 94.3%; * *p* = 0.039) was observed as well [53]. [*Main method of analysis (Student’s *t*-test).]

#### 3.2.2. Mild Cognitive Impairment

The 2019 study by Kobayashi et al. included 117 older adults (50–80 years) and analyzed the impact of *Bifidobacterium breve* A1 on cognitive dysfunction associated with neurodegeneration. Patients received two capsules daily (>1 × 10^10^ CFU) for 12 weeks. The researchers evaluated cognitive function at the beginning and conclusion of the study using the MMSE and the Neuropsychological Status (RBANS), two extensively utilized assessments for measuring cognitive impairment and neuropsychiatric function. The findings indicated substantial enhancements in the results of both assessments among the subjects who received the *Bifidobacterium breve* A1 treatment. Specifically, the most significant benefits were observed in the memory and language subscales of the RBANS, thereby underscoring the probiotic’s impact on critical cognitive domains. Moreover, a statistically significant discrepancy was detected between the treatment group and the placebo group with respect to the alterations in scores at the conclusion of the 12-week period (*p* < 0.05). Notably, no substantial disparities were identified between groups at the baseline, thereby suggesting that the observed outcomes were attributable to the treatment itself [51]. [*Main method of analysis (Student’s *t*-test).]

Another study conducted by Asaoka et al. in 2022 analyzed the role of *Bifidobacterium breve* MCC1274 (A1) in the treatment of older adults with chronological age-associated MCI. The study involved 115 patients (65–88 years) who received 2 × 10^10^ CFU daily for 24 weeks. The MMSE scores improved (<25) compared to the placebo group (“orientation in time” and “writing” subscales). A comprehensive analysis revealed a substantial increase in functional connectivity between brain regions associated with memory and cognition, as demonstrated by functional magnetic resonance imaging (fMRI) (*p* < 0.05). In the probiotic-treated group, there was a tendency to suppress the progression of brain atrophy in some subjects (volume of interest Z-score ≥ 1.0). Specifically, a significant intergroup difference was observed in the changes from the baseline of the GM (gray matter atrophy in the whole brain) extent score (*p* = 0.013) [54]. [*Main method of analysis (Student’s *t*-test).]

The study carried out by Fei et al. in 2023 analyzed the use of a probiotic combination, including different strains of *Lactobacillus* and *Bifidobacterium*, in 42 volunteer participants (over 60 years of age). Patients took a (>2 × 10^10^ CFU/g) probiotic daily for 12 weeks. The results showed that cognitive function and sleep quality were improved: MMSE (24.75 ± 2.47); Montreal Cognitive Assessment Scale—MoCA (22.05 ± 2.14 vs. 20.10 ± 1.45); and Pittsburgh Sleep Quality Index—PSQI (5.35 ± 2.78 vs. 8.40 ± 1.76; *p* < 0.001). In addition to the observed clinical improvements, the treated group exhibited a decrease in plasma levels of malondialdehyde, a marker of oxidative stress (*p* = 0.03), and an increase in the activity of antioxidant enzymes, such as catalase (*p* = 0.02). These findings suggest a protective effect on oxidative stress pathways [68]. [*Student’s *t*-test and analysis of variance (ANOVA) were used for comparative analyses.]

#### 3.2.3. Oxidative Stress

Valentini et al. in 2015 [54] conducted a study analyzing the impact of VSL#3^®^ (*Bifidobacterium infantis* DSM 24737, *Bifidobacterium longum* DSM 24736, *Bifidobacterium breve* DSM 24732, *Lactobacillus acidophilus* DSM 24735, *Lactobacillus delbrückii* ssp. bulgaricus DSM 24734, *Lactobacillus paracasei* DSM 24733, *Lactobacillus plantarum* DSM 24730, and *Streptococcus thermophilus* DSM 24731) in 62 patients (65–85 years). VSL#3^®^ was administered daily (two capsules) for 56 days. VSL#3^®^ had no significant impact on the glucose and cholesterol levels; however, VSL#3^®^ did reduce the Erythrocyte Sedimentation Rate (ESR) (*p* = 0.05), indicating a decrease in systemic inflammation. Treatment with VSL#3^®^ resulted in a favorable alteration in the composition of the intestinal microbiome, marked by a substantial increase in *Bifidobacterium* and *Lactobacillus* spp. This observation could offer a partial explanation for the observed anti-inflammatory effects.

The study revealed that this particular arm demonstrated a notable elevation in serum folate levels (*p* = 0.007) and serum vitamin B12 (*p* = 0.001). Additionally, plasma homocysteine levels exhibited a noteworthy decline (*p* = 0.001). These biochemical changes suggest a potential reduction in oxidative stress, as elevated homocysteine is a known risk factor for oxidative damage and inflammation. Additionally, both diet with and without VSL#3^®^ were correlated to an increase in the activity of glutathione-S-transferase, an enzyme involved in detoxification processes and protection against oxidative stress [54]. These findings collectively indicate that the interventions may help mitigate oxidative stress, likely through enhanced antioxidant defenses and improved nutrient status. [*Chi-squared test and Mann–Whitney U-test were used for biochemical parameters; ANOVA and ANCOVA were used for differences in inflammatory parameters before and after dietary interventions.]

In conclusion, the evidence in human studies suggests that the use of *Bifidobacterium infantis* or *Bifidobacterium longum* subsp. infantis, in combination with other strains, improved BDNF levels, reduced cortisol and ESR—Erythrocyte Sedimentation Rate—levels, and increased folate and vitamin B12 levels. Moreover, the administration of *Bifidobacterium breve* MCC1274 (A1) showed an improvement in cognitive function (MMSE and RBANS test) and significant modifications in the GM (gray matter atrophy in the whole brain) extent score [51,52,53,68]. In the study conducted by Valentini et al. in 2015, an increase in glutathione-S-transferase activity was found to be associated with reactive oxygen species—ROS—neutralization [54].

### 3.3. Animal Studies

Regarding the studies in animal models, a grand total of 12 studies were selected. Of these, 10 focused on AD [55,56,57,58,59,60,61,62,63,64] (*n* = 10) and 2 focused on PD [65,66] (*n* = 2). All of them addressed the intervention with *Bifidobacterium infantis* or *Bifidobacterium longum* subsp. infantis or *Bifidobacterium breve* to ameliorate the degeneration associated with AD or PD.

#### 3.3.1. Alzheimer’s Disease

Kobayashi et al. carried out a study in 2017 about the impact of *Bifidobacterium breve* A1 in an AD model with male (10-week-old) ddY mice. The probiotic was administered daily for 10 days (1 × 10^9^ CFU in 0.2 mL, beginning 2 days prior to Aβ injection). The outcomes of this study have revealed that *Bifidobacterium breve* A1 has a protective effect against cognitive decline induced by Aβ and inhibits the expression of genes altered by the Aβ protein in the hippocampus. Furthermore, this probiotic and acetate demonstrated a partial alleviation of behavioral impairment in the experimental group (*p* < 0.05, †† *p* < 0.01 vs. Sham) * *p* < 0.05, and ** *p* < 0.01 vs. Aβ (+)) [55]. [*One-way analysis of variance followed by Student’s *t* or Mann–Whitney U post hoc tests.]

The research conducted by Mehrabadi and Sadr, in 2020, used an AD model—Wistar rat with an Aβ1-40 intra-hippocampal injection (*n* = 50)—and administered a 2 mL probiotic mixture composed of *Lactobacillus reuteri*, *Lactobacillus rhamnosus*, and *Bifidobacterium infantis* (10^10^ CFU daily for 10 weeks). The Aβ-treated animals exhibited a longer latency compared to the control and Sham groups during the MWM—Morris water maze—training phase (*p* < 0.001). Probiotic administration was associated with enhanced spatial memory and learning in the Aβ-treated animals, compared to the controls (*p* < 0.01). Administration of the probiotic mixture showed a significant decrease in the level of malondialdehyde—MDA—compared to the Aβ-treated group (*p* < 0.001) and a reduction in the levels of inflammation biomarkers IL-1β (*p* < 0.01) and TNF-α (*p* < 0.01) [56]. [*ANOVA followed by post hoc Tukey’s test were used for comparative analyses.]

In 2021, Zhu et al. conducted a study analyzing the impact of five strains of *Bifidobacterium breve* individually (1 × 10^9^ CFU/mL) in 63 male AD model-C57BL/6J mice (8 weeks old), daily for 6 weeks. In this research, *Bifidobacterium breve* NMG and CCFM1025 use resulted in significant improvements in alternation behavior and increases in total arm entries in the Morris water maze. Nevertheless, the administration of the other three *Bifidobacterium breve* strains did not result in an improvement in working memory. In contrast, the administration of CCFM1025, XY, and WX significantly reduced the accumulation of Aβ1-42 in the hippocampus (control vs. model: # *p* < 0.05 by unpaired Student’s *t*-test; * *p* < 0.05 and ** *p* < 0.01 by one-way ANOVA for all groups) [57]. [*One-way analysis of variance (ANOVA) or the Kruskal–Wallis test with post hoc comparison.]

Abdelhamid et al. (2022) carried out research using the 2-month-old C57BL/6J mouse model of AD (*n* = 40). The animals were treated with *Bifidobacterium breve* MCC1274 (1 × 10^9^ CFU/mL) five times/week for four months vs. Sham. There was a substantial reduction in the soluble Aβ1-42 levels in the hippocampal extracts of the probiotic mice compared to the Sham group; the levels of the p-Akt and p-GSK-3β proteins were markedly elevated in the hippocampus of the probiotic group compared to the Sham group (* *p* < 0.05); and the administration of *Bifidobacterium breve* MCC1274 resulted in a notable elevation in the protein levels of SYT and syntaxin and exhibited a tendency to increase the levels of SYP and PSD-95 in the hippocampal preparations (** *p* < 0.01) [58]. [*Two-tailed unpaired Student’s t-test (for normally distributed variables).]

Another study conducted by Abdelhamid et al. in 2022, with a similar methodology, addressed the impact of *Bifidobacterium breve* MCC1274 (A1) in an AD model with 3-month-old App knock-in (KI) mice (AppNL-G-F) (*n* = 52). *Bifidobacterium breve* A1 (1 × 10^9^ CFU in 0.2 mL, administered 2 days prior to the injection of Aβ) was administered daily for 10 days. The probiotic group demonstrated a significantly enhanced exploration time for the novel object in comparison to the familiar object, thereby improving memory impairment. Furthermore, the discrimination index (DI) was observed to be higher in the probiotics group in comparison to the Sham group. *Bifidobacterium breve* MCC1274 supplementation has been demonstrated to suppress Aβ fibril formation, with a significant upregulation of ADAM10 and PS1 observed in the hippocampus. In contrast, no change was observed in the AβPP and BACE1 levels (*p* < 0.05) [59]. [*Main method of analysis (Student’s *t*-test).]

Deng et al., in 2022, conducted a study analyzing the effect of 2.5% inulin (3.6 g/kg/day) compared with a synbiotic (4.1 g/kg/day), daily for 2 months on an APP transgenic mouse line (J20 and Wild type (WT)). This synbiotic contained the inulin prebiotic and Vitalon Probiotics (VP) composition. The results showed that the level of Aβ1-42 was significantly decreased in the experimental group compared with the control mice; the synbiotic treatment significantly reduced the TNF-α levels (* *p* < 0.05; *** *p* < 0.001; and **** *p* < 0.0001) [60]. [*Differences between multiple means were assessed by one-way or two-way ANOVA.]

Zhu et al. conducted a study in 2022 analyzing the impact of Bifidobacterium breve CCFM1025 in 40 male adult AD model-C57BL/6J mice (8 weeks old). The animals received the probiotic (1 × 10^9^ CFU/mL) alone or in combination with an enriched environment—EE—daily for 6 weeks. The combination of an EE and *Bifidobacterium breve* CCFM1025 resulted in enhancements in working memory as demonstrated by the Y-maze test. Additionally, the accumulation of hippocampal Aβ1-42 was considerably decreased in the EE-treated groups, with EE + *Bifidobacterium breve* CCFM1025 (control vs. model: # *p* < 0.05, ## *p* < 0.01, ### *p* < 0.001, and #### *p* < 0.0001) [61]. [*In order to conduct a parametric analysis of the observed differences between groups, a one-way analysis of variance (ANOVA), with the Holm–Sidak test or Student’s *t*-test, was employed. In the case of data exhibiting non-parametric properties, a variance analysis approach was utilized, employing the Kruskal–Wallis test, which was followed by Dunn’s test or Welch’s *t*-test.]

Another study conducted by the research group of Zhu et al. (2022) evaluated the impact of *Bifidobacterium breve* CCFM1025 in 24 adult male AD model-C57BL/6J mice (8 weeks old). The animals received the probiotic (1 × 10^9^ CFU/mL) alone or Sham-sterilized 10% skim milk, daily for 6 weeks. The results have shown that the use of *Bifidobacterium breve* CCFM1025 in the model group restored the level of L-tyrosine and tryptophan (*p* < 0.05); in comparison to the control group, up to 36 different metabolites were significantly altered in the hippocampal tissue of the experimental model (*p* < 0.05) vs. CCFM1025; and the levels of phenylalanine and L-glutamine in the hippocampus were restored (* *p* < 0.05; ** *p* < 0.01) [62]. [*The main method of analysis was Principal Component Analysis (PCA) for all the samples, along with the Pearson correlation coefficient for the QC samples.]

The study conducted by Zhu et al. [63] in an AD model with male mice (16 weeks old) addressed the impacts of *Bifidobacterium breve* HNXY26M4 vs. Sham. The animals received *Bifidobacterium breve* HNXY26M4 (1 × 10^9^ CFU/mL) daily for 12 weeks and the results indicated that the use of *Bifidobacterium breve* HNXY26M4 led to a drastic reduction in the levels of Aβ1−42 compared to those in APP/PS1 mice. It also reduced the neuroinflammation processes, oxidative damage, and synaptic disruption in the APP/PS1 mice brains. The acetate and butyrate levels in *Bifidobacterium breve* HNXY26M4-treated mice were increased, and there was a more significant rise in acetate compared to butyrate (*p* < 0.01 and *** *p* < 0.001) [63]. [*One-way ANOVA.]

Recent research conducted by Zhu et al., in 2024, investigated the efficacy of administering *Bifidobacterium breve* HNXY26M4 (1 × 10^9^ CFU/mL) daily to male adult AD model-C57BL/6J mice (8 weeks old) (*n* = 40), alone or administered with an EE, for 6 weeks. The results indicated that this probiotic reduces cognitive impairment and modulates glutamine metabolism, improving brain function. Specifically, mice that received only environmental enrichment (EE, ADEE) or EE combined with *Bifidobacterium breve* treatment (AD+BBEE) had significantly lower concentrations of Aβ1-42 in the hippocampus than AD-SE mice (* *p* < 0.05, ** *p* < 0.01, and *** *p* < 0.001) [64]. This indicates that both environmental enrichment and the combination of EE with *Bifidobacterium breve* treatment are effective in reducing Aβ1-42 levels, a marker associated with Alzheimer’s disease pathology. It is important to note that while EE alone was effective, the addition of *Bifidobacterium breve* did not diminish the beneficial effects, suggesting that the probiotic may complement the environmental intervention rather than acting independently. The combined treatment may offer a synergistic effect, enhancing overall outcomes. [*Student’s *t*-test and analysis of variance (ANOVA) were used for comparative analyses.]

In conclusion, the results obtained from AD model animal studies have demonstrated that *Bifidobacterium breve* (A1)-CCFM1025 has the capacity to prevent cognitive dysfunction, improve memory impairment, and ameliorate behavioral deficits [51,52,65]. Furthermore, A1 has been shown to suppress Aβ fibril formation, and the protein levels of p-GSK-3β and p-Akt in the hippocampus have been found to be significantly increased [51,53,65]. Furthermore, *Bifidobacterium breve* (A1)-CCFM1025 has been demonstrated to restore levels of tryptophan and L-tyrosine, as well as the serum metabolite phenylalanine and L-glutamine, in the hippocampus [57].

The studies that addressed the treatment with *Bifidobacterium breve* HNXY26M4 concluded that this probiotic produced a significant decrease in the Aβ1-42 level in the hippocampus and ameliorated neuroinflammation, oxidative damage, and synaptic impairment in the brain, especially in areas associated with memory and learning, including the hippocampus [63,64]. In the same vein, the studies that administered a mixture probiotic or individual strains of *Bifidobacterium breve* showed that the use of *Bifidobacterium infantis*, *Bifidobacterium longum* subsp. Infantis, or *Bifidobacterium breve* improves the mnesic function, decreases the MDA levels [68], reduces the Aβ1-42 level in the hippocampus, and reduces the TNF-α levels [57,60].

#### 3.3.2. Parkinson’s Disease

Ishii et al. carried out research in 2021 and *Bifidobacterium breve* A1 was evaluated in a PD model with male C57BL/6 mice (7–8 weeks old) (*n* = 156). The probiotic was administered vs. Sham, using the same method: *Bifidobacterium breve* MCC1274 (A1) (1 × 10^9^ CFU/mL), daily for 4 days. In this study, the results in PD mice indicated that *Bifidobacterium breve* A1 prevented the decrease in spine density and kept it at the same level as in the control group (* *p* < 0.01) vs. Control + Saline († *p* < 0.05): Between MPTP + Saline and MPTP + *Bifidobacterium breve* A1, *Bifidobacterium breve* A1 had no effects on the levels of hippocampal cAMP-adenylyl cyclase (AC)–cyclic AMP in the PD and control mice (* *p* < 0.01 vs. Control + Saline) [65]. [*Tukey’s post hoc test or Kruskal–Wallis analysis and Mann–Whitney U tests were conducted based on homoscedasticity analyses using Levene’s test.]

In 2022, Li et al. [66] conducted a study which addressed the impact of *Bifidobacterium breve* CCFM1067 (10^9^ CFU/200 μL saline) used daily between days 8 and 41. The authors used a PD model with male C57BL/6 mice (6 weeks old) (*n* = 40). This study concluded that *Bifidobacterium breve* CCFM1067 reduced the motor deficits: Both the L-DOPA and *Bifidobacterium breve* CCFM1067 treatments significantly showed a reduction in MPTP-induced motor impairments measured in the PT—pole test (F (2,21) = 56.94; *p* < 0.0001), NBT—narrow-beam test (F (2,21) = 33.72; *p* < 0.0001), and RTR—rotarod—test (F (2,18) = 21.99; *p* < 0.0001). Bifidobacterium breve CCFM1067 showed a reduction in striatal TNF-α (F (2,9) = 91.49; *p* < 0.0001), IL-1β (F (2,9) = 29.53; *p* < 0.0001), and IL-6 (F (2,9) = 24.61; *p* = 0.0002) [69]. [*The D’Agostino–Pearson test, Student’s unpaired *t*-test, or one-way analysis of variance (ANOVA) with Dunnett’s test were employed to assess the statistical significance of differences between two or multiple datasets. The Mann–Whitney test or Kruskal–Wallis test with Dunn’s test were utilized to evaluate the statistical significance of the differences between two or multiple datasets, respectively] [66].

The evidence suggests that the use of *Bifidobacterium breve* MCC1274 (A1) exerts a neuroprotector function through the prevention of the spine density reduction in Parkinson’s disease models. The result about the absence of modification in the hippocampal cAMP-adenylyl cyclase (AC)–cyclic AMP levels is interesting [65]. The use *of Bifidobacterium breve* CCFM1067 showed improvement in motor impairments and reduced the increase in striatal TNF-α [66].

## 4. Discussion

The primary aim of this systematic review was to identify and compile the most significant findings derived from research conducted in human and animal models investigating the effects of *Bifidobacterium infantis*, *Bifidobacterium longum* subsp. Infantis, or *Bifidobacterium breve*, either alone, in conjunction, or in combination with specific strains of these or other species, on the improvement in symptomatology and cellular damage associated with neurodegenerative disease. The results obtained indicated that these probiotics improve motor symptoms and neuropsychiatric impairments and reduce neuronal damage alone and in combination with other strains. In comparison, *Lactiplantibacillus* (*Lactobacillus*) *plantarum* has also shown significant potential in alleviating neurological symptoms. Thus, *Lactiplantibacillus plantarum* can improve neuropsychiatric disorders, sleep disorders, and autonomic dysfunction in patients with neurological conditions [70,71]. Furthermore, *Lactiplantibacillus* plantarum has been found to enhance cognitive function and mitigate neuronal damage when used in combination with other treatments, such as memantine [72]. Both *Bifidobacterium* strains and *Lactiplantibacillus plantarum* exhibit beneficial effects on the gut–brain axis, supporting their use as psychobiotics for improving both motor and neuropsychiatric symptoms [40].

Analyzing the impact of *Bifidobacterium infantis*, *Bifidobacterium longum* subsp. Infantis, or *Bifidobacterium breve*, alone, in conjunction, or in combination with other strains on neurodegenerative diseases, it was observed that both human and animal studies have mainly focused on AD, PD, oxidative stress, and MCI, as shown in Table 1.

The administration of *Bifidobacterium* in the context of AD has been shown to exert neuroprotective effects, which are associated with significant changes in the serum profile and in outcomes related to memory and learning. Regarding the serum profile, an increase in key biomarkers such as the brain-derived neurotrophic factor, implicated in neuroplasticity and cognitive function, was observed, along with a reduction in inflammatory markers, such as interleukin-1β and cortisol levels. This indicates a decrease in the systemic inflammatory response and oxidative stress. In addition, modulations were identified in essential metabolites such as acetate and butyrate, as well as in amino acids such as phenylalanine and L-glutamine, critical elements in neuronal metabolism and the integrity of the gut–brain axis. In this vein, interventions involving *Bifidobacterium* have been associated with substantial enhancements in behavioral assessments pertaining to memory and cognitive function. These interventions have been observed to result in a strengthening of processes, such as information acquisition and retention capacity. These observations have been made in both animal models and clinical trials [53,55,56,57,58,59,60,61,62,63,64,65,66,70,71].

Addressing the use of probiotics in AD, recent studies have shown a beneficial effect of *Lactiplantibacillus plantarum* in reducing symptomatology, either in isolation or in conjunction with other strains. Similarly, other strains of *Bifidobacterium* (such as *Bifidobacterium lactis* and *Bifidobacterium longum*) have demonstrated favorable outcomes in both human and animal studies [73,74].

In a recent study, Shi et al. (2022) investigated the impact of *Bifidobacterium longum* BB68S on cognitive function in older adults without cognitive impairment. The results indicated that the administration of this probiotic strain improved cognitive functions and favorably regulated the participants’ GM [75].

Other studies have been conducted that explore the relationship between altered intestinal microbiota and the presence of AD. A study by Xia et al., published in 2023, investigated the GM in patients with AD compared to healthy controls. The results of the study suggest a link between altered gut microbiota, particularly the presence of *Bacteroides fragilis*, and the pathogenesis of AD [76]. This bacterium could hinder the capacity of microglia to clear amyloid-beta from the brain, resulting in an augmented amyloid plaque burden [77]. Regarding other *Bifidobacterium* strains, previous studies have demonstrated beneficial effects in AD, including a 2019 study showing improvements in the serum profile and learning after probiotic treatment (*Bifidobacterium bifidum* or *Bifidobacterium longum*), though without memory function enhancement [78].

Regarding PD, two studies analyzed the impact of different strains of *Bifidobacterium breve*. Specifically, the administration of *Bifidobacterium breve* MCC1274 (A1) showed protective effects by preventing the reduction in spine density [65]. Moreover, the use of *Bifidobacterium breve* CCFM1067 showed improvements in motor symptomatology and a reduction in striatal TNF-α [66]. It is widely accepted that damage to the striatum is responsible for the motor symptoms observed in PD [79,80,81].

Concerning the use of probiotics in PD, previous studies have shown that the administration of *Lactobacillus* and *Bifidobacterium* improved motor symptoms. Among others, the research addressing the use of *Lactobacillus acidophilus*, *Lactobacillus fermentum,* and *Lactobacillus reuteri* showed neuroprotective effects and a reduction in oxidative stress. In addition, this genus is useful for the improvement in neuropsychiatric symptomatology associated with PD for its ability to modulate mood, or the quality of sleep, among others [82].

Regarding mild cognitive impairment, it was observed that the use of *Bifidobacterium breve* MCC1274 (A1) improved memory deficits. However, in the study conducted by Kobayashi et al., no significant differences were found in the pre–post comparison of the MMSE and RBANS scores [51]. Asaoka et al. [52] found that this probiotic improved GM levels compared to the control group, suggesting better gut health and reduced dysbiosis. In addition, in the 2023 study conducted by Fei et al. [68], a probiotic mixture containing various strains of *Lactoplantibacillus* and *Bifidobacterium* was used. The study reported significant improvements in the MMSE and MoCA scores, as well as in sleep quality measured by the PSQI (Pittsburgh Sleep Quality Index) [68].

Previous studies have analyzed the impact of probiotics on MCI. For instance, *Lactiplantibacillus plantarum* has been shown to improve memory function, reduce neuroinflammation, and improve synaptic dysfunction [83,84], and *Lactobacillus plantarum* DR7 has demonstrated improvements in memory and neuroprotective effects [69].

In a 2020 study conducted by Xiao et al., *Bifidobacterium breve* was administered to older adults with MCI for 16 weeks. The results showed that those who took *Bifidobacterium breve* had significantly increased scores on the MMSE and RBANS compared to the control group [85]. In their study, Yang et al. in 2020 [76] used a probiotic mixture consisting of *Bifidobacterium lactis*, *Lactobacillus casei*, *Bifidobacterium bifidum*, and *Lactobacillus acidophilus* on animals for 12 weeks. The results showed a significant improvement in memory deficits and a decrease in previous cognitive dysfunction. Furthermore, an improvement in synaptic function and neuronal functioning were observed [86].

In recent years, an increasing number of studies have examined the effects of probiotic use on oxidative stress. For example, the study conducted by Valentini et al. (2015) found an increase in glutathione-S-transferase activity after the probiotic use [54]. This protein plays a role in reducing ROS, regulating membrane permeability, and participating in the synthesis of proteins, DNA, and RNA, among others [87,88].

Lin et al. in 2022 [89] conducted an animal study on the effects of a probiotic mixture containing *Bifidobacterium animalis* subsp. infantis BLI-02, *Bifidobacterium breve* Bv889, *Bifidobacterium bifidum* VDD088, *Bifidobacterium animalis* subsp. lactis CP-9, and *Lactobacillus plantarum* PL-02. The study found that this probiotic mixture had a neuroprotective effect and increased butyrate levels, which are directly associated with inflammatory and oxidative processes [89]. However, it is imperative to acknowledge that elevating butyrate levels may engender a range of effects that are not invariably associated with the attenuation of inflammation. Some of these effects may not be salutary, contingent upon the prevailing physiological conditions. For instance, in a study on pediatric obesity, oral butyrate supplementation was observed to result in adverse effects such as nausea and headaches in two patients during the first month of intervention [90].

In 2020, Ton et al. conducted a study analyzing the use of a prebiotic *kefir* mixture composed with different strains of *Acetobacter*, *candida Lactobacillus*, *Enterococcus*, and *Leuconostoc* in patients with oxidative stress and dementia. The study findings indicate an enhancement in cognitive function through mechanisms related to cell damage, organ inflammation, and oxidative stress [91].

Li et al. (2022) conducted a recent study that evaluated the effects of Bifidobacterium breve CCFM1067 in a mouse model of MPTP-induced Parkinson’s disease. The results demonstrated that the administration of this probiotic strain regulated the GM and alleviated the motor dysfunction associated with Parkinson’s disease, suggesting a neuroprotective effect. These findings provide a compelling rationale for the utilization of psychoactive probiotics, such as *Bifidobacterium breve* CCFM1067, in the prevention and treatment of Parkinson’s disease [66].

The results reported in this research have shown that probiotic intervention may improve symptoms associated with cognitive impairment in neurodegenerative diseases, such as memory, spatial orientation, learning, and level of anxiousness, among others. Additionally, *Bifidobacterium* probiotics have demonstrated neuroprotective properties and the ability to modulate several metabolic processes associated with neurodegeneration. While acknowledging certain limitations, these data serve as a valued step for addressing degenerative processes and neurodegenerative diseases.

In the context of probiotic strain combinations, a number of studies have examined potential synergies that may augment therapeutic outcomes in various pathological conditions. For instance, a study by Li et al. (2019) examined the combination of *Lactobacillus acidophilus* and *Bifidobacterium animalis* subsp. lactis, revealing that the amalgamation of these strains exhibited a considerably more pronounced anti-inflammatory effect in human colon cells (HT-29) compared to the individual strains alone. This finding suggests a potential for the utilization of this combination in the treatment of inflammatory bowel diseases, which could have implications for disorders involving neuroinflammation [92]. A recent study by Nian et al. (2023) demonstrated that the combination of *Akkermansia muciniphila* and *Bifidobacterium bifidum* protected against the development of non-alcoholic fatty liver disease (NAFLD) in mice by regulating the expression of FXR and the intestinal microbiota. While the study focused on liver pathology, it underscores the potential of these combinations to address metabolic dysfunctions, which could be related to neurodegenerative diseases [93].

These studies suggest that combinations of *Bifidobacterium* with other bacterial spp., such as *Lactobacillus* or *Akkermansia*, could have significant therapeutic potential in various inflammatory and metabolic conditions. However, further research is necessary to fully elucidate their mechanisms of action and applicability in neurological disorders.

## 5. Limitations and Future Research

One limitation that should be noted is the small number of registered clinical trials. The identification of biological markers is an active research field, although the use of metabolomics is a recent development. Nonetheless, the data collected in this systematic review are relevant. These data provide a solid and rigorous empirical basis for identifying starting points for future research.

It should be noted that the databases consulted contain a limited number of full free articles. It is important to consider the possibility of validating results with studies that are not accessible to the scientific community.

It is important to consider another limitation, which is that the evaluated interventions are heterogeneous in terms of the administered dose and intervention duration. Additionally, some probiotics are used alone while others are used in combination with different strains, species, and even families, making it difficult to generalize the beneficial results obtained from their administration. Specifically, this lack of evidence on its effect when used alone affects *Bifidobacterium infantis*. Further study of the mechanisms of action of each administered probiotic is necessary.

This systematic review represents the initial effort to elucidate and accentuate the impact of *Bifidobacterium infantis* and *Bifidobacterium breve*, alone or in combination, on neurodegenerative diseases. The collected empirical evidence supports the development of interventions that incorporate the use of analyzed *Bifidobacterium* strains to improve motor, cognitive, and behavioral symptoms related to these pathologies, as well as to delay related neurodegenerative processes. Nonetheless, it is premature to consider these results as definitive support for specific therapeutic applications. Although dietary supplements containing these strains are commercially available, their effectiveness in clinical settings requires further scientific evidence. This evidence should be increased through longitudinal and controlled studies, including clinically relevant biomarkers and reproducible protocols.

## 6. Practical Applications

This review emphasizes the pivotal role of the intestinal microbiota in the development and progression of neurodegenerative diseases, observing that imbalances in its composition are associated with disorders such as Alzheimer’s and Parkinson’s. Alteration of the microbiota has the capacity to compromise cellular homeostasis and promote neuroinflammation, a critical factor in neurodegeneration. In this regard, probiotics, particularly certain strains of *Bifidobacterium*, have demonstrated encouraging results in ameliorating dysbiosis and enhancing cognitive function in animal models and, to a lesser extent, in human studies. While the modulation of the gut–brain axis can generate benefits, there may also be results or undesirable effects, such as possible interactions with other treatments or unexpected alterations in the GM. Furthermore, the return of the gut microbiota to its normal state may vary between individuals, necessitating a personalized approach.

The therapeutic potential of probiotics suggests the ability to improve gut health, reduce inflammation, and potentially slow the progression of neurodegenerative diseases. However, it is essential to recognize that clinical evidence is still limited and that current studies cannot affirm with certainty the long-term effectiveness and safety of these treatments. Strict adherence protocols and the use of clinically relevant biomarkers are essential to advance research to understand the underlying mechanisms and evaluate the effects of probiotic intervention.

## 7. Conclusions

The results from both human and animal studies indicate that *Bifidobacterium infantis*, *Bifidobacterium longum* subsp. infantis, and *Bifidobacterium breve* probiotic strains may improve neuropsychiatric symptoms, enhance cognitive functions, and reduce neuroinflammation and oxidative stress markers. Specifically, the administration of these *Bifidobacterium* strains was associated with improvements in memory, spatial orientation, and learning abilities, alongside a decrease in anxiety levels and enhanced motor function. These findings support the hypothesis that targeted probiotic interventions can positively modulate gut–brain interactions, potentially offering a neuroprotective effect that delays the progression of neurodegenerative diseases. Therefore, incorporating these probiotics into therapeutic protocols presents a promising avenue for enhancing the quality of life for individuals suffering from conditions such as Alzheimer’s and Parkinson’s disease.
